# A Rare Case of a Prostatic Abscess Secondary to a Mycoplasma hominis Infection

**DOI:** 10.7759/cureus.31491

**Published:** 2022-11-14

**Authors:** Abhijeet Jagtap, Krishanu Das, Mhd Firas Safadi

**Affiliations:** 1 Department of Pathology, St James University Hospital, Leeds Cancer Center, Leeds, GBR; 2 Department of Urology and Andrology, Bahrain Specialist Hospital, Manama, BHR; 3 Department of Visceral, Thoracic and Vascular Surgery, University Hospital Carl Gustav Carus, Technische Universität Dresden, Dresden, DEU

**Keywords:** pcr multiplex, multiplex pcr, transurethral drainage, transrectal ultrasound scan, mycoplasma infections, mycoplasma hominis, acute prostatitis, prostate surgery, prostatic abscess, prostate abscess

## Abstract

Mycoplasma hominis is one of the pathogenic organisms that may cause prostatitis with the development of a prostatic abscess in very rare cases. A 57-year-old man presented with lower urinary tract symptoms and low-grade fever. The transabdominal ultrasonography showed prostate enlargement suggesting acute prostatitis. The patient was started on empiric antibacterial therapy with fluoroquinolones. The urine and semen cultures showed no bacterial growth. A few days later, the patient presented again with symptoms progression and acute urinary retention. The transrectal ultrasound revealed diffuse calcifications and intraprostatic fluids. The computed tomography of the abdomen and pelvis showed a large abscess in the prostate with a periprostatic inflammatory reaction. While all bacterial cultures were negative, the multiplex polymerase chain reaction (PCR) test revealed a Mycoplasma hominis infection. The patient was managed with transurethral drainage. After six months of follow-up, the patient was free of symptoms and the repeat PCR study confirmed clearance of the Mycoplasma infection.

## Introduction

Prostate abscesses are pus collections in the prostate gland, which usually develop on the background of acute bacterial prostatitis. Due to the widespread use of antibiotics in bacterial prostatitis, prostate abscesses are currently uncommon in clinical practice, as they only constitute 0.5% of all urologic diseases [1].

Prostatic abscesses present with lower urinary tract symptoms that may be associated with systemic symptoms such as fever and chills [2]. Most abscesses are caused by Enterobacteriaceae, with other pathogenic organisms being less common [3]. Many cases are refractory to antibiotic treatment and require surgical drainage [4].

In this report, we present an unusual case of prostate abscess caused by Mycoplasma hominis. We describe the initial presentation of the patient, the clinical progression of the case, the used diagnostic modalities, and the clinical management.

## Case presentation

A 57-year-old man presented to the urology clinic with lower urinary tract symptoms including urgency, dysuria, and frequency of urination for a few days. He also noticed low-grade fever without chills. No hematuria or flank pain was reported. The patient denied nausea, vomiting, or other gastrointestinal symptoms. The last unprotected sexual contact was six weeks before the presentation. Otherwise, the patient has no significant medical or surgical history, takes no regular medications, and has no previous history of urinary tract infections.

The clinical examination was unremarkable. The abdominal examination was normal with no tenderness on abdominal palpation or paravertebral percussion. In the urogenital examination, there were no urethral discharge, scrotal changes, or testicular abnormalities. The rectal examination revealed mild tenderness in the prostate. The transabdominal ultrasonography showed prostate enlargement to about 73 ml.

Based on the clinical findings, a diagnosis of acute prostatitis was made. Urine and semen specimens were sent for bacterial culture. The patient was started on a fluoroquinolone (ciprofloxacin) as empiric antibacterial therapy, an alpha blocker, and a non-steroidal anti-inflammatory medication.

**Figure 1:**
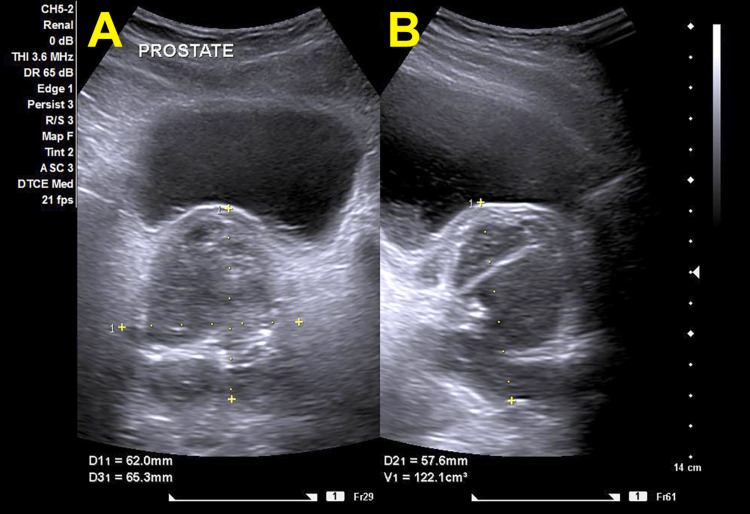
Transabdominal ultrasound examination of the prostate. Both the axial (A) and the sagittal (B) sections show an inhomogeneous and irregular enlargement of the prostate with an estimated size of 122.1 ml. No abnormalities can be identified in the urinary bladder.

After a few days, the patient presented with acute urinary retention. He reported persistent symptoms with progressive voiding difficulty and incomplete emptying of the urinary bladder. The patient was managed with the insertion of a urinary catheter. The previously obtained urine and semen cultures were available at this point and showed no bacterial growth. The rectal examination revealed extreme tenderness in the prostate without fluctuation. A repeat ultrasound examination showed an increase in prostate volume to 122 ml without definite abscess formation (Figure 1). The therapy was switched to wide-spectrum parenteral antibiotics with meropenem.

**Figure 2:**
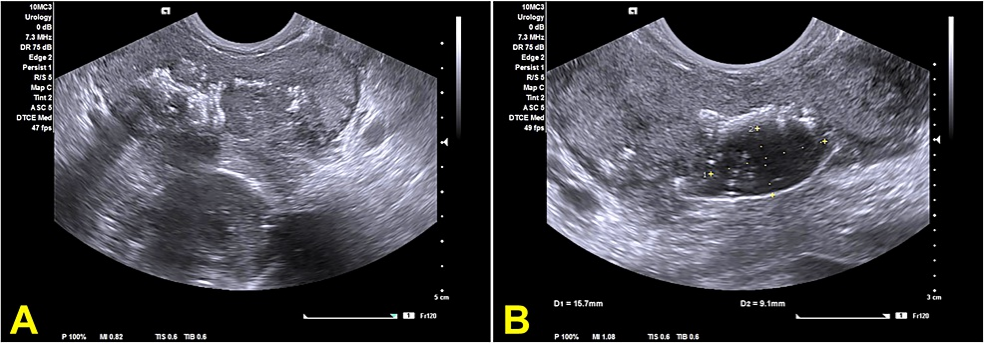
Transrectal ultrasound scan of the prostate. (A) The section shows a diffuse enlargement of the prostate gland with scattered calcifications. (B) The section shows a hypoechoic area within the prostate gland with a subcapsular leak of fluid. The findings are consistent with a prostatic abscess.

On the next day, the patient complained of increasing perineal pain with greenish urethral discharge. Urethral swabs were sent for culture and multiplex polymerase chain reaction (PCR) test. Due to the progression of symptoms, we organized a transrectal ultrasound, which revealed a further prostatomegaly with a volume of about 150 ml as well as diffuse calcifications and intraprostatic fluids in the left lobe (Figure 2).

**Figure 3:**
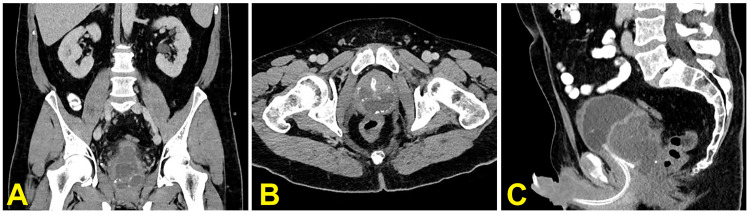
Computed tomography of the abdomen and pelvis with oral and intravenous contrast medium in the venous phase. The coronal (A), axial (B), and sagittal (C) views of the prostate show diffuse enlargement, scattered calcifications, and a complex abscess structure with intraprostatic fluids. Image C shows the course of the urinary catheter up to the urinary bladder, with the mass of the abscess extending into the small pelvis and pushing the urinary bladder upwards and forwards. Note also the diffuse periprostatic inflammatory reaction in images A and C.

For further evaluation, computed tomography of the abdomen and pelvis was performed. This showed a large abscess in the prostate measuring about 6.7x5.4x5.0 cm with periprostatic inflammatory changes (Figure 3). 

The repeated urine cultures for aerobics and anaerobics yielded again negative results. The multiplex PCR revealed a Mycoplasma hominis infection. One gram of azithromycin was immediately administered, and a course of oral doxycycline was started. We made the indication for transurethral drainage of the abscess.

In the cystoscopy, the large abscess was identified in the left lobe of the prostate proximal to the verumontanum. The abscess pocket was deroofed with drainage of a large amount of pus. An extensive lavage of the abscess cavity was performed. The patient showed postoperative improvement of the symptoms and was able to void successfully after catheter removal.

**Figure 4:**
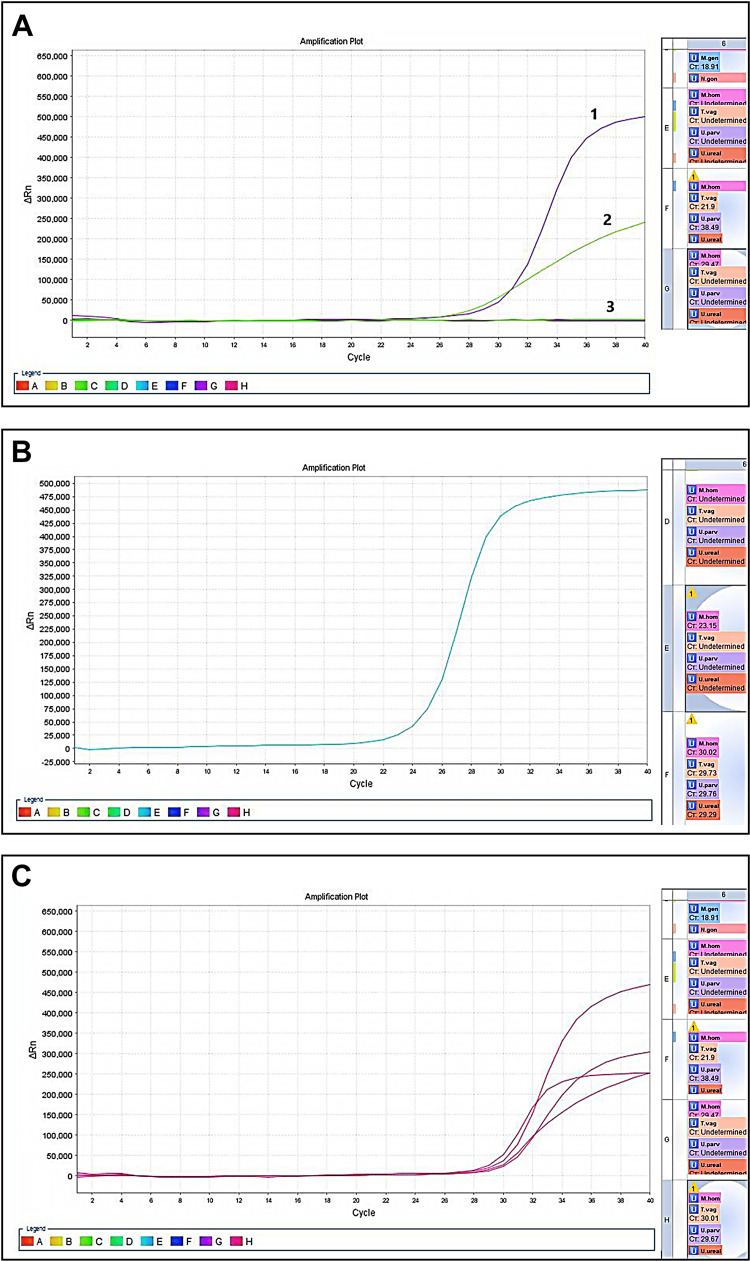
Polymerase chain reaction (PCR) amplification plots. (A) Multiplex real-time PCR carried out on the prostatic tissue scrapings. Curve 1 depicts the amplified DNA target of Mycoplasma hominis (M. hominis). Curve 2 depicts an amplified internal (endogenous) control, confirming the proper isolation technique pre-PCR. Curve 3 is a flat unamplified negative control confirming the absence of contamination. The window G on the right reveals a threshold cycle (Ct) value of 29.47 for M. hominis only; other microbial DNAs were not detected. (B) Multiplex real-time PCR carried out on the urethral swab. Note that the amplification for M. hominis starts at a Ct value of 23.15 (window E on the right side). (C) Multiplex real-time PCR carried out on the prostatic tissue and urethral swab. All the curves depicted here are amplified targets of known positive control samples indicating a valid probe and PCR reaction.

The culture of the pus from the abscess cavity as well as tissue scrapings of the prostatic abscess yielded again no bacterial growth. The multiplex PCR of the prostatic tissue scrapings from the cavity confirmed the Mycoplasma hominis infection (Figure 4).

In the last follow-up visit six months postoperatively, the patient reported a complete resolution of symptoms. The repeat ultrasound examination showed a normal-size prostate, no residual collections, and no significant post-void residue. A repeat PCR study confirmed clearance of the Mycoplasma infection.

## Discussion

Prostatic abscesses are one of the possible complications of prostatitis and may present a diagnostic dilemma [1]. It may be difficult to differentiate them from other diseases that present with lower urinary tract symptoms, especially in the early stages. The diagnosis should be suspected in patients with persistent fever or worsening symptoms despite antibiotic therapy [2]. Severe tenderness or the presence of fluctuation on rectal examination is a common finding. In advanced cases, they may present with local compression and acute urinary retention, as in our case. Since a wide range of organisms can be responsible for prostatitis and subsequent abscess formation, laboratory specimens should be collected before starting antibiotics. These may include urinalysis, expressed prostatic secretions, or semen cultures [5].

Before the advent of modern antibiotic therapy, 75% of prostatic abscesses were attributable to Neisseria gonorrhea, and the mortality rate was between 6-30% [2]. Presently, Enterobacteriaceae, especially Escherichia coli, are the predominant pathogenic culprit in prostatic abscesses. Less common isolates include Klebsiella species, Proteus mirabilis, Enterococcus faecalis, and Pseudomonas aeruginosa [3]. Methicillin-resistant Staphylococcus aureus was also isolated from prostatic abscesses in elderly patients, immunocompromised individuals, patients with indwelling urinary catheters, following healthcare exposure, and after genitourinary surgery [6].

Hematogenous dissemination from a septic focus in the respiratory, digestive, or urinary tracts or soft tissue infections is also possible, albeit very rare. The organisms detected in such cases include Staphylococcus aureus, Mycobacterium tuberculosis, Escherichia coli, and Candida species. With the early diagnosis and the availability of good antibiotics, the current mortality rates of prostatic abscess range from 1% to 16% [5].

Mycoplasmas are the smallest free-living microorganisms. Due to the lack of a cell wall, they do not take Gram stain and they are difficult to grow in the laboratory with routine microbial culture methods [7]. The absence of the cell wall renders them resistant to antibiotics that target cell wall synthesis such as beta-lactams. Mycoplasma hominis is an opportunistic saprophyte residing in the human lower urogenital tract and it is commonly implicated in sexually transmitted genitourinary tract infections [7,8]. It was isolated from the semen of patients with chronic prostatitis [9] and was also reported in patients with prostate cancer [10].

However, prostatic abscesses caused by Mycoplasma hominis have not yet been reported. In our case, repeated urine and semen cultures did not reveal any bacterial growth, including anaerobic organisms. The multiplex PCR test performed on the urethral swab and prostatic tissue confirmed the infection with Mycoplasma hominis. The history of the patient implies a sexually-transmitted infection, which is well-described for Mycoplasma hominis [8], although it was not clear why he developed this infection in the absence of immunodeficiency or chronic diseases.

Detection techniques for Mycoplasma hominis include DNA probes, enzyme-linked immunoassay (ELISA), and polymerase chain reaction (PCR). DNA probing is expensive and not readily available, while ELISA detects the antigens/antibodies mainly in the blood days after the initial infection and has relatively low sensitivity and specificity [11,12]. PCR, in particular real-time PCR, is considered a rapid test with high sensitivity and specificity for detecting the DNA of these organisms [12]. It allows the detection of as little as 1000 copies of microbial DNA in urogenital specimens, with a negative predictive value of more than 99% [12,13].

The technique used in our laboratory employs a standardized nucleic acid extraction protocol based on magnetic beads. It applies a multiplex real-time PCR for all urogenital swabs, abscesses, and urine specimens for amplification. The test uses a commercially available probe with a two-tube multiplex for the detection of common sexually transmitted micro-organisms including Mycoplasma hominis, Mycoplasma genitalium, Ureaplasma species, Trichomonas, Chlamydia, and Neisseria species.

The PCR is carried out by an automated thermal cycler (Applied Biosystems ABI 7500 Real-Time PCR System, Distribio Laboratory Instruments, Luxemburg). The amplification curves are obtained and calculated relative to the baseline and control targets. In the presented case, specimens from prostatic tissue scrapings and urethral swabs repeatedly showed an amplification pattern for Mycoplasma hominis (Figure 4A, 4B). The cut-off values for exponential amplification are called threshold cycle (Ct) values and they are auto-calculated based on the standardization of baseline and control targets. According to the repetitive amplification results from two separate specimens, the infection with Mycoplasma hominis was confirmed and the diagnosis of the abscess was based on the clinical and radiological findings.

Every patient with acute prostatitis who does not respond to initial antibiotic therapy should have further imaging to rule out abscess formation [14]. Transrectal ultrasound is the initial imaging modality of choice. It usually demonstrates ill-defined hypoechoic areas within an enlarged or distorted prostate gland [15]. Computed tomography is another diagnostic modality that can show well-defined areas of low attenuation. Additionally, it detects a potential extra-prostatic spread of the abscess, particularly into the ischiorectal fossa and perineum [14]. Magnetic resonance imaging is not used widely for diagnosis, possibly due to the rarity of the condition and the availability of other imaging methods. The usual characteristics of prostatic abscesses include a hypointense signal on T1 and a hyperintense signal on T2 [16].

All patients with a prostatic abscess should be treated with antibiotics. Two weeks of treatment may suffice for small abscesses [1]. Ultrasound-guided aspiration transrectally or transperineally is easy to perform, has low morbidity, and can be repeated in case of failure or incomplete drainage [15]. Transurethral deroofing is a more appropriate therapy for abscesses larger than 30 ml, multifocal lesions, recurrent cases, or abscesses that are not completely drained under ultrasound guidance, as it ensures better drainage of the abscess cavity with the enhancement of early recovery [4]. The surgical indication in our case was made based on the clinical course and the abscess progression on imaging. We chose a transurethral approach to ensure complete drainage and relieve the obstruction. Open transperineal drainage is rarely required, usually for abscesses extending beyond the prostate and penetrating the pelvic floor [5].

## Conclusions

This report presents a rare case of acute prostatitis with Mycoplasma hominis with development of a prostatic abscess. The patient presented with lower urinary symptoms and fever, as the clinical presentation of abscesses induced by Mycoplasma hominis does not differ from those resulting from other organisms. Prostatic infection with Mycoplasma should be suspected in any patient with negative cultures for common pathogens as well as failure to respond to antibiotic therapy. PCR test was used to confirm the diagnosis and it is considered the most sensitive and specific diagnostic modality for prostatic infections with Mycoplasma hominis.

Transrectal ultrasound and computed tomography are important diagnostic adjuncts in prostatic abscesses. They help delineate the size and the extension of the abscess and aid in planning the appropriate surgical treatment. The abscess size was less than 7 cm in this patient with no involvement of the adjacent structures, which made it amenable to transurethral drainage. Large or locally-advanced lesions may require an open transperineal approach to ensure adequate drainage.
